# A multiscale seasonal examination of the risk of harm to seabirds from vessels based on co‐occurrence in Alaskan waters

**DOI:** 10.1111/cobi.70115

**Published:** 2025-08-20

**Authors:** Kelly Kapsar, Benjamin K. Sullender, Katherine J. Kuletz

**Affiliations:** ^1^ Department of Fisheries and Wildlife Michigan State University East Lansing Michigan USA; ^2^ Kickstep Approaches Anchorage Alaska USA; ^3^ School of Environmental and Forest Sciences University of Washington Seattle Washington USA; ^4^ Audubon Alaska Anchorage Alaska USA; ^5^ U.S. Fish and Wildlife Service Anchorage Alaska USA

**Keywords:** Arctic, light pollution, marine traffic, North Pacific, seabird, seabird–vessel collisions, vessel traffic, wildlife disturbance, Ártico, ave marina, colisiones entre aves marinas y buques, contaminación lumínica, Pacífico Norte, perturbación de fauna, tráfico de buques, tráfico marítimo

## Abstract

Alaska's seascape supports globally significant seabird populations, including vulnerable and threatened species, and hosts economically important commercial fisheries and marine transportation corridors. Seasonal patterns of seabird movements and vessel traffic create a complex landscape of risk, defined as high levels of co‐occurrence (overlap) between seabirds and vessels. Areas of high overlap increase risk of detrimental impacts, such as exposure to artificial light from ships, bycatch, behavioral disturbance, collision, and oil spills. To investigate this risk landscape, we combined satellite‐based automatic identification system (AIS) vessel traffic data (2015–2022) with at‐sea, ship‐based seabird observation data from the North Pacific Pelagic Seabird Database (2006–2022). We used these data to analyze seabird–vessel overlap from June through August (summer) and September through November (fall). Presence of both vessels and birds was highest in summer, presenting a greater overall exposure of seabirds to vessel‐related impacts than in fall. This risk in both seasons was associated with vessel traffic corridors, such as Unimak Pass and the Bering Strait. When only nighttime vessel traffic was considered, risk of disturbance or interaction was higher in fall than in summer north of ∼60° N latitude. Across seasons, regions of highest risk varied by focal taxonomic group. *Aethia* auklets were most exposed in the northern Bering and Chukchi Seas, and *Ardenna* shearwaters and northern fulmars (*Fulmarus glacialis*) were most exposed in Unimak Pass. Overall, our findings provide an essential foundation for management decision‐making to reduce risk of vessel‐related injury, contamination, disturbance, displacement, and mortality for marine birds and other wildlife. The heterogeneous distribution of risk across taxa and the persistent spatial concentration of high‐risk areas together require targeted, area‐based mitigation approaches for effective conservation.

## Abstract (es)

Análisis estacional multiescala del riesgo de daño de buques para las aves marinas basado en la coocurrencia en aguas de Alaska

**Resumen:**

El paisaje marino de Alaska alberga poblaciones de aves marinas de importancia mundial, incluidas especies vulnerables y amenazadas, y acoge pesquerías comerciales y corredores de transporte marítimo de gran importancia económica. Los patrones estacionales de los movimientos de las aves marinas y el tráfico marítimo crean un complejo panorama de riesgos, definido como altos niveles de coocurrencia (superposición) entre las aves marinas y los buques. Las zonas de alta superposición aumentan el riesgo de impactos perjudiciales, como la exposición a la luz artificial de los buques, las capturas accidentales, las perturbaciones del comportamiento, las colisiones y los derrames de petróleo. Para analizar este panorama de riesgos, combinamos los datos del tráfico marítimo del sistema de identificación automática (SIA) por satélite (2015‐2022) con los datos de observación de aves marinas en el mar desde buques de la Base de Datos de Aves Marinas Pelágicas del Pacífico Norte (2006‐2022). Utilizamos estos datos para analizar la superposición entre aves marinas y embarcaciones de junio a agosto (verano) y de septiembre a noviembre (otoño). La presencia tanto de embarcaciones como de aves fue mayor en verano, lo que supuso una mayor exposición general de las aves marinas a los impactos relacionados con los buques que en otoño. Este riesgo en ambas estaciones se asoció con corredores de tráfico marítimo, como el paso de Unimak y el estrecho de Bering. Cuando sólo consideramos el tráfico marítimo nocturno, el riesgo de perturbación o interacción fue mayor en otoño que en verano al norte de los 60° de latitud norte. A lo largo de las estaciones, las regiones de mayor riesgo variaban según el grupo taxonómico focal. Las alcas *Aethia* eran las más expuestas en el norte de los mares de Bering y Chukchi, y las pardelas *Ardenna* y los fulmares boreales (*Fulmarus glacialis*) eran las más expuestas en el paso de Unimak. En general, nuestros hallazgos proporcionan una base esencial para la toma de decisiones de gestión destinadas a reducir el riesgo de lesiones, contaminación, perturbación, desplazamiento y mortalidad de las aves marinas y otros animales silvestres relacionados con los buques. La distribución heterogénea del riesgo entre los taxones y la persistente concentración espacial de las zonas de alto riesgo requieren, en conjunto, enfoques de mitigación específicos y basados en el área para una conservación eficaz.

## INTRODUCTION

As climate change disrupts ecosystems, effective conservation of migratory wildlife requires assessing when and where these changes are taking place and identifying emerging risks. Globally, seabirds have one of the highest proportions of threatened species among all bird groups (Croxall et al., 2012). Subarctic and Arctic migrating seabirds, in particular, face dual climate‐mediated pressures from documented major shifts in marine ecosystem function (Duffy‐Anderson et al., 2019; Huntington et al., 2020; Kuletz, Gall, et al., 2024; Moore & Stabeno, 2015) and altered distribution of anthropogenic impacts (Kapsar et al., 2023). Vessel activity is associated with seabird bycatch from commercial fisheries (Clay et al., 2019; Croxall et al., 2012; Dietrich et al., 2025; Žydelis et al., 2013), disturbance (Dehnhard et al., 2020; Dias et al., 2019; Schwemmer et al., 2011), and interference with seabird navigation that can lead to collision events (Coleman et al., 2022; Merkel & Johansen, 2011). Although published data are sparse, light pollution from vessels traveling at night or in inclement weather can also interfere with seabird navigation at sea, particularly during seasonal migrations (Rodríguez et al., 2019; Ronconi et al., 2015). Light pollution may have larger effects at high latitudes, where fall migration periods overlap with low‐light conditions and increased storm activity (Gjerdrum et al., 2021; Merkel & Johansen, 2011). In addition to these direct seabird–vessel interactions, increased vessel activity also elevates the risk of secondary impacts, such as chronic exposure to contaminants, displacement of prey, and catastrophic oil spills (Huntington et al., 2015; Lieske et al., 2019; Piatt et al., 1990). Seabird dependence on marine habitats and evidence of detrimental interactions make them an ideal taxonomic group for examination of the conservation impacts of vessel traffic.

**FIGURE 1:**
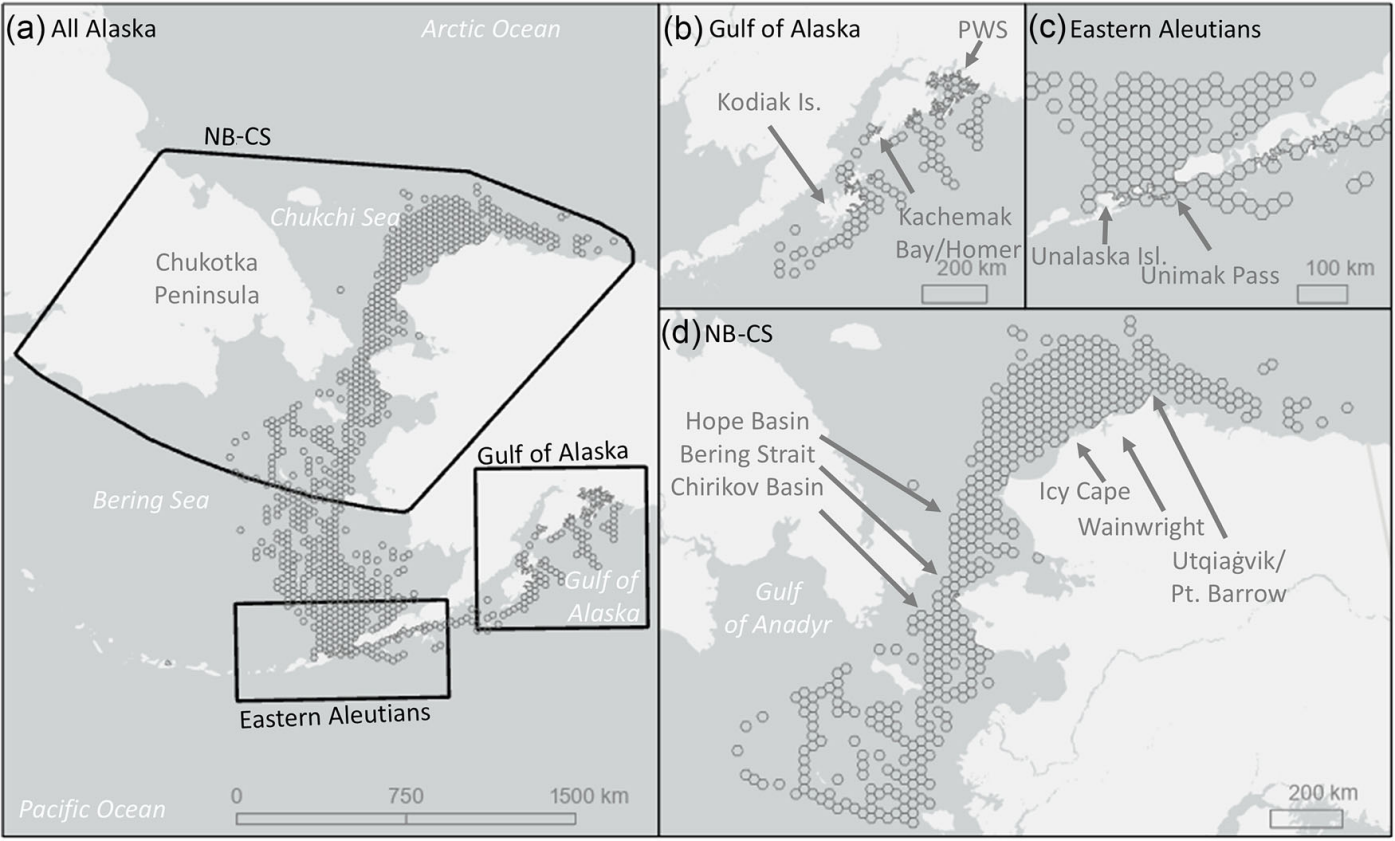
Areas (hexagons) with sufficient seabird survey effort and available vessel traffic data for analyses of seabird–vessel overlap in (a) all of Alaska, (b) Gulf of Alaska (GOA), (c) eastern Aleutian Islands (EAI), and (d) northern Bering and Chukchi Seas (NB‐CS).

The United States contains the largest number of breeding seabird species in the world (Croxall et al., 2012), most of which rely on Alaskan waters. Alaska's oceans extend from the Gulf of Alaska to the western Aleutians, and north into the Bering, Chukchi, and Beaufort Seas (Figure 1). Nutrient‐rich waters of the North Pacific upwell onto the continental shelves of the Gulf of Alaska and the Bering Sea, fostering an array of marine life (Grebmeier et al., 2006; Santora et al., 2018; Sigler et al., 2011), which in turn support both globally significant commercial fisheries and seasonal migrations of approximately 40 million seabirds (Kroodsma et al., 2018; Santora et al., 2018; U.S. Fish and Wildlife Service MBM, 2009).

Alaskan waters also contain critical shipping routes for the transportation of people and resources across the Pacific and the Arctic (Arctic Council, 2009). Concurrent with strong economic incentives (Brigham, 2011), the longer ice‐free season in the northern portion of this region has and will continue to increase vessel traffic activities, particularly fishing vessels and liquified‐natural gas (LNG) tanker movements along the Russian coast and Bering Strait (Kapsar et al., 2023). For populations of seabirds already stressed by climate change, the potential consequences of increased vessel traffic could exacerbate multiple environmental challenges.

Despite the importance of Alaskan waters for both birds and maritime commerce, there has not been an overarching, area‐wide analysis of seabird–vessel overlap. Research has been limited to taxon‐ or area‐specific estimates of risk of seabird–vessel interactions (Dietrich et al., 2009; Humphries & Huettmann, 2014; Renner & Kuletz, 2015; Renner et al., 2018). For instance, in an analysis of risk in the Aleutian Archipelago, Renner and Kuletz (2015) found high seabird–vessel overlap in the area of Unimak Pass. Observations of direct mortality events from seabird–vessel interactions (known as bird storms) have documented dozens to hundreds of individual bird mortalities in a single incident, often during inclement weather (Dick & Donaldson, 1978; Merkel & Johansen, 2011; NMFS, 2020a; Ryan et al., 2021).

No previous work prioritized conservation issues for Alaska's oceans on a range‐wide scale. The diversity of vessel traffic and seabird species, strong seasonal differences in the distributions of both, and variability in the total amount of nighttime vessel exposure all indicate the need for a comprehensive assessment to prioritize conservation and management actions. To address this information gap, we pursued 3 primary objectives: quantify the seasonal risk of seabird exposure to vessel traffic in Alaska's oceans (Objective 1), delineate high‐risk areas for focal taxonomic groups in regions of elevated or changing vessel activity (Objective 2), and determine whether nighttime vessel traffic, when considered separately, changes the distribution of risk (Objective 3).

To meet these research objectives, we examined broadscale seasonal patterns of co‐occurrence for seabird taxa susceptible to detrimental vessel interactions (impacts) for all Alaskan marine waters and for 3 focal regions. We organized our analyses into 2 parts. First, we created a risk index to evaluate the spatial and seasonal patterns of exposure for seabirds across Alaskan waters. This method allowed for comparison between focal regions and identification of the highest risk focal regions for each taxonomic group. Second, we reanalyzed risk with only data from vessels transmitting their locations at nighttime and compared the total high‐risk areas identified using all vessel data with the risk generated by vessels traveling at night. The nighttime traffic analysis could help parse out how the spatiotemporal distribution of risk might change with respect to particular threats, such as nighttime vessel lights.

## METHODS

### Description of study area

Our analyses covered over 527,000 km^2^ of Alaskan waters, including the western Gulf of Alaska, Aleutian Islands, eastern Bering Sea, and the eastern Chukchi Sea (Figure 1). These 3 large marine ecosystems were included in our analyses of seabird–vessel overlap for all of Alaska (177.5°–141.5° W and 51°–73° N). Due to a combination of low seabird survey coverage, seabird densities (Kuletz et al., 2019; Wong et al., 2014), and vessel traffic, we did not include the Beaufort Sea. Based on results for all of Alaska, we conducted finer scale analyses of 3 focal regions identified as containing regularly used vessel traffic routes and important seabird habitats: northcentral Gulf of Alaska (NGA), eastern Aleutian Islands (EAI), and northern Bering Sea and eastern Chukchi Sea (NB‐CS) (Figure 1a). The NGA and EAI are subarctic seas, and the NB‐CS includes subarctic and arctic waters (Bellamy, 2010).

The NGA focal region extends across the south‐central Alaskan coast (157°–145° W and 55°–61° N). Adjoining lands support most of Alaska's human population and major marine ports (Figure 1b). Vessel activity includes cargo transport, oil and gas production and transport, commercial fishing, ferries, and tourism. There are large commercial fisheries in and around Kodiak Island and a well‐established network of ferry routes throughout Prince William Sound (PWS), which is also the corridor for oil transport from Valdez to the Gulf of Alaska. Lower Cook Inlet is the entry to the Port of Anchorage, which handles oil and gas transport and the majority of Alaska's inbound cargo traffic from the greater Gulf of Alaska (McDowell Group, 2020). A variety of commercial fisheries also cover Cook Inlet, PWS, and the NGA shelf (Woodby et al., 2005). The NGA hosts more than 65 bird species, many of which migrate seasonally to, from, or within the focal region (Hunt et al., 2005; Springer, 2006). Approximately 35 species, including murres, puffins, and kittiwakes, return each year to breed along rocky coastal cliffs, with over 1120 colonies across the greater Gulf of Alaska coastline (Springer, 2006; Stephensen & Irons, 2003).

In the Aleutian Islands, the EAI focal region spans 84,000 km^2^ (170°–158° W and 53°–56° N) (Figure 1c). This region serves as a gateway for international vessel traffic along the North Pacific Great Circle Route. The easternmost and largest passage of this route between the NGA and Bering Sea is Unimak Pass, a 40‐km‐wide passage connecting trade between North America and Asia. Each year, thousands of vessels transit through Unimak Pass transporting cargo (Sullender et al., 2021). The EAI focal region also includes the Bering Sea port of Dutch Harbor, the highest volume fishing port in the United States (NMFS, 2020b). Additionally, fishing vessels travel the region targeting multiple commercial fishing stocks managed by the National Oceanic and Atmospheric Administration (primarily in the U.S. Exclusive Economic Zone) and the Alaska Department of Fish and Game (primarily within 3 nautical miles of shore), including 5 of the 12 largest seabird colonies in Alaska and hundreds of smaller colonies (Stephensen & Irons, 2003; Woodby et al., 2005). The Aleutian Islands also serve as important breeding sites for an estimated 500,000 seabirds. The region also provides foraging grounds for millions of migratory seabirds that arrive to feed during the northern summer (Byrd et al., 2005; Jahncke et al., 2005; Renner & Kuletz, 2015).

The NB‐CS focal region extends from 60° N to 73° N latitude (177° to 141° W longitude) and is oceanographically and biologically connected with water and fauna flowing northward through the ∼80‐km‐wide Bering Strait (Sigler et al., 2011, 2017) (Figure 1d). The Bering Strait is the only corridor for maritime access to the Arctic from the Pacific Ocean and is thus the gateway for Arctic shipping routes (Arctic Council, 2009; Waloven et al., 2023). As duration and extent of sea ice formation have changed (Markus et al., 2009; Stabeno & Bell, 2019), this region has experienced more vessel traffic in the last decade (Kapsar et al., 2023; Thomson et al., 2022). The NB‐CS focal region is ecologically rich, with approximately 60 seabird species recorded at some phase of the open‐water season (Gall et al., 2022; Kuletz et al., 2019). Colonies with several million birds are located on a few larger islands or mainland cliffs, with smaller colonies scattered along barrier islands and the coastal tundra (Kuletz et al., 2015; Stephensen & Irons, 2003). In summer, high densities of seabirds tend to occur near colonies (Piatt & Springer, 2003) but can also occur offshore where prey species are aggregated, such as in the Anadyr Current in the northern Bering Sea or Hanna Shoal in the Chukchi Sea (Gall et al., 2022; Kuletz et al., 2019). The Bering Strait itself serves as an important migratory corridor for seabirds during summer and fall. The northern Bering polynyas and open leads are also important habitat for some species of sea ducks from fall through spring (Lovvorn et al., 2015). Among the focal regions, the NB‐CS has the greatest change in the amount of daylight throughout the year. In the northern portion of the NB‐CS region, the sun does not set at all between May and August, but during fall, the amount of daylight rapidly declines until the polar night begins in mid‐November, and the sun does not rise until late January.

### Vessel traffic data

To quantify vessel activity, we acquired automatic identification system (AIS) data for 8 years (2015–2022) across our study area. Collection of vessel activity data is required by the International Maritime Organization for all vessels >300 gross tons on an international voyage, all cargo vessels over 500 gross tons, and all passenger vessels (IMO, 2002; Robards et al., 2016). The United States has additional requirements for AIS on all commercial vessels over 65 feet in length (Taconet et al., 2019).

We cleaned the AIS data following the procedures in Kapsar et al. (2022). In brief, we removed transmissions from erroneous locations, stationary beacons, other nonvessel entities, and vessels with invalid identifiers; created a lattice of 25‐km (625 km^2^) hexagons (hereafter, cells) over the entire study area; spatially intersected cells with a land‐cover map to obtain accurate measurements of the area of coastal cells; and intersected the grid with all AIS transmissions and calculated vessel activity statistics for each cell.

For each vessel in each 24‐h period, we calculated the total number of hours of vessel activity as well as the total hours of activity at night. The time of day at which a transmission occurred was determined by the latitude, longitude, and time stamp fields to calculate the solar position at the precise time and location of each transmission with the maptools package in R version 4.3.0 (Bivand et al., 2013; R Core Team, 2023). To account for extended twilight at high latitudes, we used the end and start of civil twilight to define nighttime. We calculated the total hours of activity as the time difference between the first and last transmission in a given day. Given that there may be multiple separate instances of daylight or nighttime in a given 24‐h period, we calculated total hours of daylight and nighttime activity based on the cumulative amount of time between transmissions during daylight and nighttime hours, respectively. We then transformed the vessel activity to the +1 log to calculate the final vessel activity value for each cell in each season as

(1) $$V_{i,s}=\log\left(\mathrm{hour}s_{i,s}+1\right),$$

where $V_{i,s}$ is the total hours of operation by ships in cell *i* during season *s* transformed to the log +1.

Given the large volume of raw data (>600 GB), we processed these data in monthly batches in parallel on Michigan State University's High Performance Computing Cluster. We then aggregated vessel activity metrics at a seasonal time scale.

### Seabird distribution data

To evaluate seabird sightings, we used at‐sea seabird survey data from the North Pacific Pelagic Seabird Database 4.0 (Drew et al., 2015, 2023). This database contains over 21 million individual ship‐based seabird observations along ∼3‐km‐long transects obtained throughout the North Pacific with standardized protocols since 1973. Surveys were typically conducted from vessels of opportunity; thus, seabird survey transect locations depended on the sampling stations and transit lines of various projects, many of which were multiyear or ongoing monitoring and research programs. In brief, for at‐sea surveys, observers used binoculars to obtain or confirm identification of a bird in a set strip transect width from a vessel traveling at ∼4–8 kts. Birds were counted continuously except for flying birds, which were counted using a snapshot method (frequency depending on vessel speed to avoid overcounting flying birds) (Gould & Forsell, 1989). Observers attempted to avoid counting birds following the vessel or entering the transect from behind. A portion of older data (7%) counted flying birds continuously. Seabird densities (birds per square kilometer) were calculated based on the area surveyed, and the centroid of each transect was used as the location of the sample. Details on the origins and protocols used for the NPPSD surveys are in Drew et al. (2023).

We filtered the seabird observation data to include only those made from 2006 to 2022 (*n* = 1.3 million birds), in order to better align with the available vessel traffic data and to maintain sufficient sample size for analyses. We accounted for a few extreme outliers in seabird observations (*n* = 7) primarily due to rare, huge flocks of *Ardenna* shearwaters by setting a maximum count of 10,000 birds per observation. All values above this number were rescaled to 10,000 prior to density calculations.

To ensure sufficient survey coverage and facilitate comparison between seasons, we excluded cells with <1% of total area surveyed in each season. To facilitate comparison across seasons, we included only cells with sufficient survey effort in both summer and fall in the final analysis (*n* = 873 cells in each season).

We calculated the final effort‐weighted seabird density values ($D_{i,t,s}$) for each taxonomic group in each cell *i* in each season *s* as follows:

(2) $$D_{i,t,s}=\log\left(\frac{\sum_{r=1}^{n}\frac{\mathrm{nob}s_{r,t}}{\mathrm{ef}f_{r}}}{n}+1\right),$$

where $\mathrm{nob}s_{r,t\;}$ is the number of individuals of taxonomic group *t* observed along transect *r*; $\mathrm{ef}f_{r}$ is the survey effort for a given transect; and *n* is the total number of transects in a given cell in a given season.

We calculated effort‐weighted seabird densities for all seabird species in aggregate (hereafter, total seabirds) and for 5 taxonomic groups susceptible to detrimental vessel interactions (impacts) (Appendices S1 & S2; Equation 2): auklets (3 *Aethia* species), shearwaters (2 *Ardenna* species), northern fulmars (*Fulmarus glacialis*), storm petrels (2 species), and sea ducks (seven species). Together, these 15 species represented 59% of total seabird observations in summer and 73% in fall in Alaska. This approach of calculating risk separately for different taxonomic groups also allowed us to account for differences in population sizes that would influence the overall impact of interactions with vessels. For example, high‐risk areas for taxonomic groups with smaller population sizes offshore (e.g., sea ducks) may be dwarfed by vessel–bird overlap with more abundant taxonomic groups (e.g., shearwaters). However, by examining sea duck observations separately, we identify areas of high risk that are specific to that group.

### Analysis of seabird–vessel overlap

We constructed 2 analyses. First, we used a continuous analysis of seabird–vessel overlap to quantify the risk of seabird exposure to vessel traffic across space and by season for the entire study area (Objectives 1 and 2). Second, we repeated the analysis with only data from vessels operating at night to determine whether there was a change in the distribution and magnitude of seasonal overlap between birds and nighttime vessel traffic (Objective 3) (workflow overview in Appendix S4). Both analyses were conducted for 2 seasons: summer (June through August) and fall (September through November). This seasonal definition encompassed the variability in seabird phenology, separating the main breeding season in the North Pacific during summer months and postbreeding migration in fall, and captured potential variability in vessel traffic, which typically is more active in summer but may increase in the far north during the fall sea ice minimum (Arctic Council, 2009; Kapsar et al., 2023).

To measure risk of exposure, we calculated the degree of overlap between seabird and vessel intensities (Halliday et al., 2022). This method relies on the assumption that increases in either seabird density or vessel traffic increase overall risk to seabirds. We measured risk on a continuous scale with a 100‐point unitless risk index. This approach allowed us to evaluate the highest risk focal regions and seasons for each taxonomic group independently. We calculated the risk index value for each unique cell, taxonomic group, and season combination ($RI_{i,t,s}$) as the product of rescaled seabird density and vessel activity metrics:

(3) $$RI_{i,t,s}=\left(\frac{V_{i,s}-\min\left(V\right)}{\mathrm{range}\left(V\right)}\times 10\right)\times \left(\frac{D_{i,t,s}-\min\left(D_{t}\right)}{\mathrm{range}\left(D_{t}\right)}\times 10\right)$$

The risk index thus fell along a 0–100 unitless scale (0, no risk; 100, greatest possible risk value). We created seasonal risk maps for each focal taxonomic group and calculated summary statistics of risk index values for each focal taxonomic group in each focal region and season. Nighttime risk was evaluated following the same methods as the previous analysis, except that for the vessel traffic we only used data from vessels transmitting at nighttime (details in “Vessel traffic data”).

## RESULTS

**FIGURE 2:**
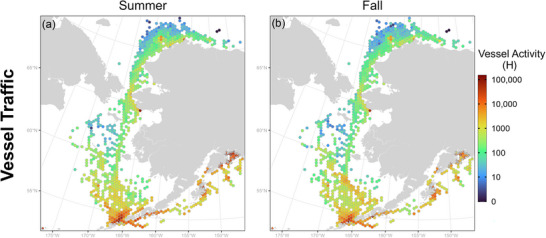
Alaska‐wide vessel traffic in (a) summer and (b) fall.

Across the entire study area, there was 38% more vessel traffic activity in summer than in fall (Appendix S3). A similar pattern held true for each of the 3 focal regions when considered separately. Despite seasonal differences in the amount of traffic, the spatial distribution of vessel traffic was broadly similar between seasons, albeit with some differences in fishing grounds in the central Bering Sea and in PWS (Figure 2). Likewise, the spatial distribution of nighttime vessel traffic did not differ substantially from daytime (Appendix S5). Areas of high vessel activity were concentrated around PWS, northeastern Kodiak Island, and the approaches to Unimak Pass. Moderate activity levels were seen near Nome and in the corridor from the Bering Strait to Point Barrow.

**FIGURE 3:**
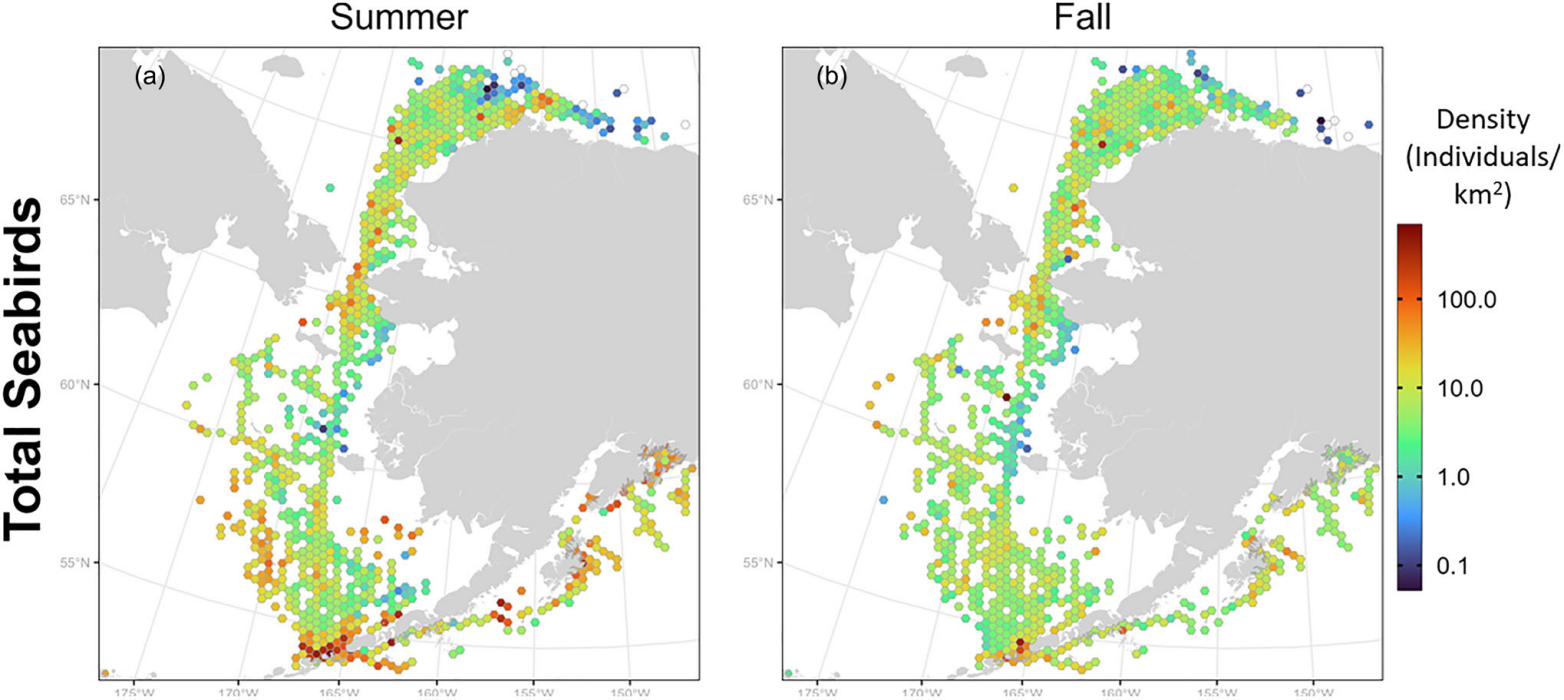
Alaska‐wide seabird density in (a) summer and (b) fall.

Seabirds were widely distributed throughout the study area in both summer and fall (Figure 3). During summer, areas of highest seabird densities included the area in and around Unimak Pass and the Bering Strait. Densities were moderately high throughout NGA, the outer shelf of the southeast Bering Sea, and throughout the eastern Chukchi Sea from Hope Basin to Point Barrow. During fall, densities were moderately high east of Kodiak Island, in a smaller area near Unimak Pass, and in scattered individual cells from the Bering Strait to Point Barrow.

**FIGURE 4:**
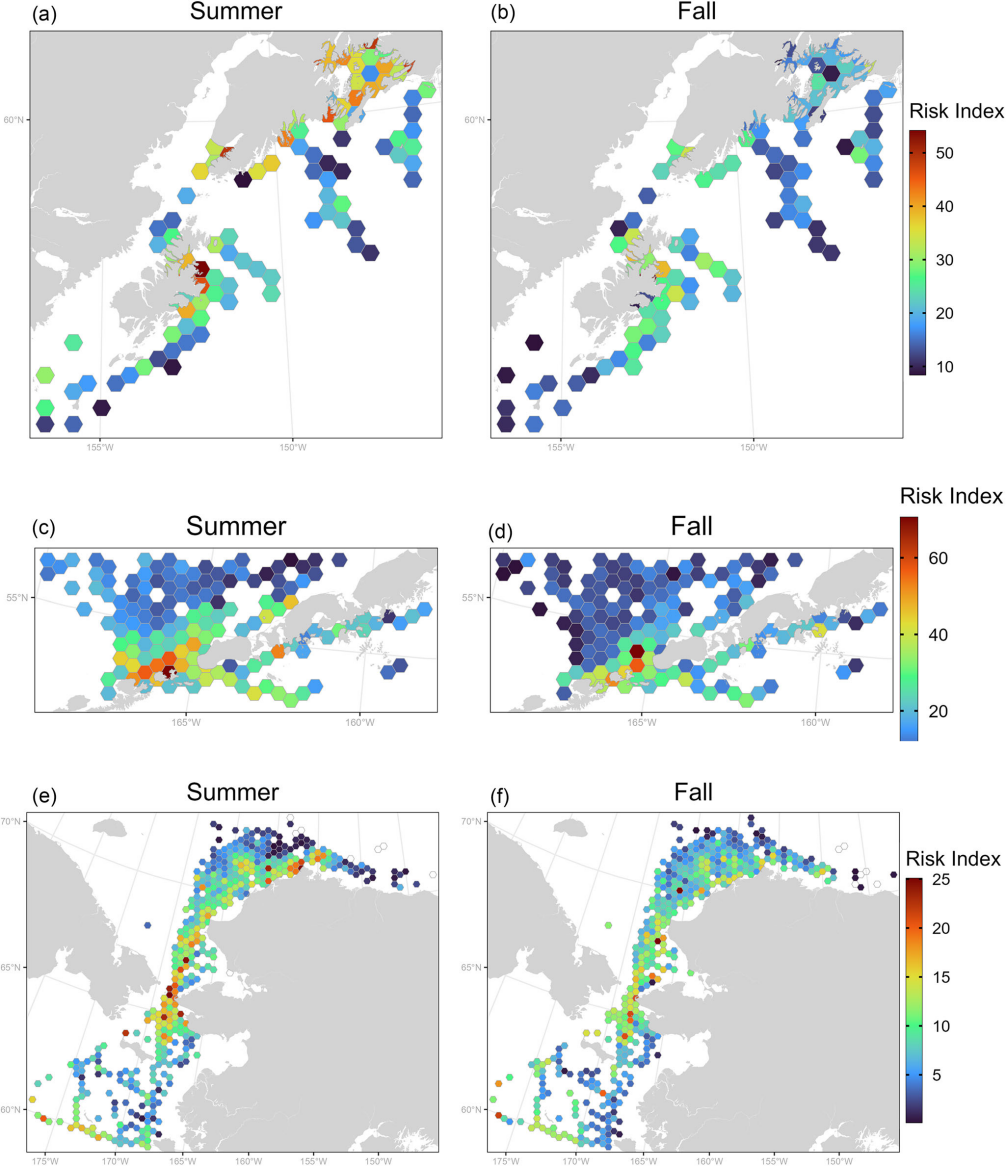
Seasonal index values of risk of seabird–vessel interactions for (a) summer in Gulf of Alaska, (b) fall in Gulf of Alaska, (c) summer in eastern Aleutian Islands, (d) fall in eastern Aleutian Islands, (e) summer in northern Bering and Chukchi Seas, and (f) fall in northern Bering and Chukchi Seas.

**FIGURE 5:**
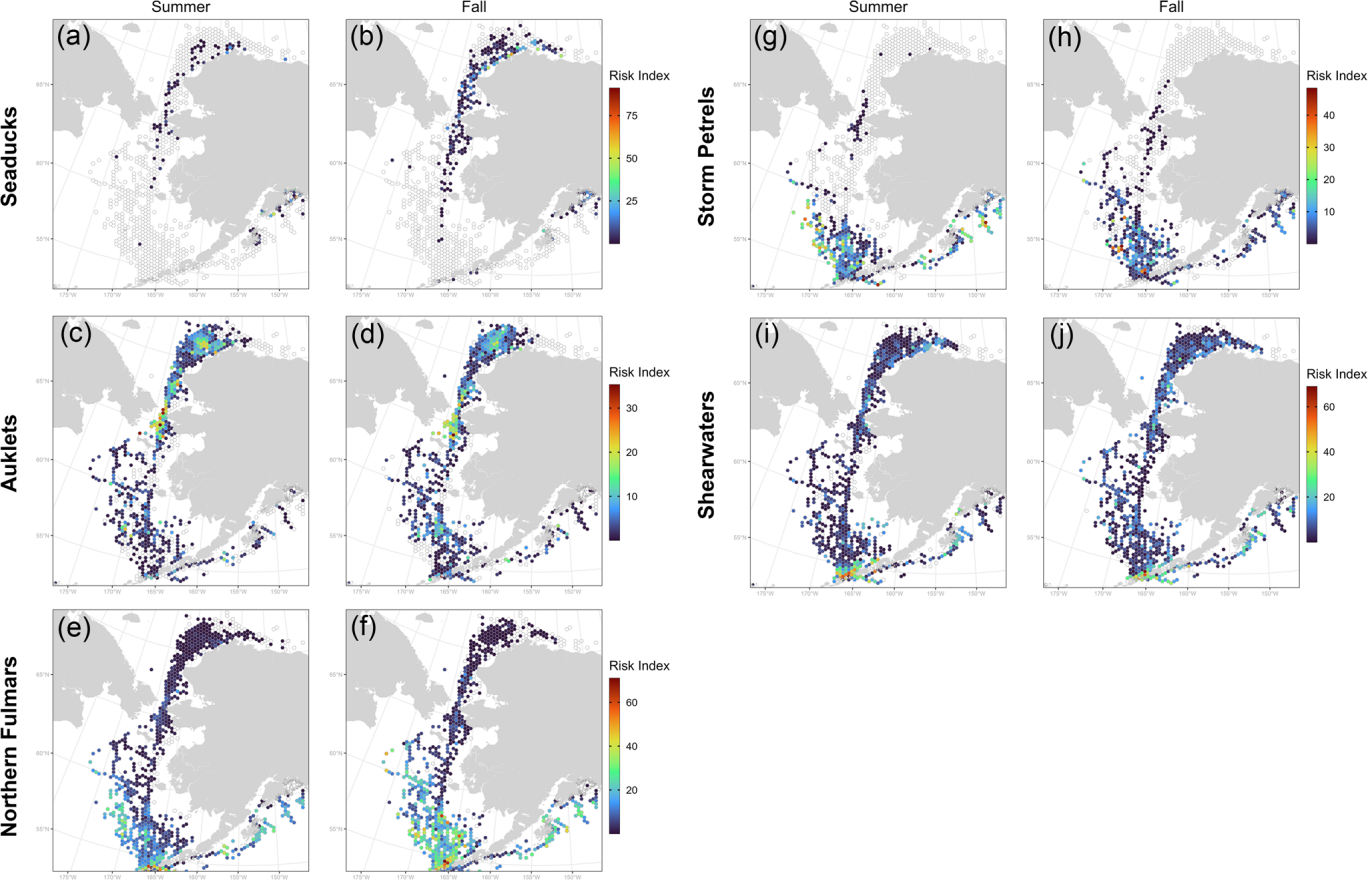
Seasonal index values of risk of seabird–vessel interactions for (a, b) sea ducks, (c, d) auklets, (e, f) northern fulmars, (g, h), storm petrels, and (i, j) shearwaters in fall (left) and spring (right).

Across all cells, mean risk index values for total seabirds were 13.9 and 13.4 for summer and fall, respectively (Table 1). Regional average risk index values were highest for the NGA (26.0 and 20.3 in summer and fall, respectively) and lowest for the NB‐CS region (8.5 and 9.3 in summer and fall, respectively) (Figure 4). The EAI had mean risk index values slightly lower than the NGA in summer but very similar in fall (21.4 and 19.7, respectively). For all of Alaska, the area with the highest risk indices for total seabirds was in and around Unimak Pass (both seasons) (Appendix S6a,b). Moderately high risk was also identified in PWS, the southern Kenai Peninsula, and northeast Kodiak, primarily in summer (Figure 5a).

**TABLE 1:** Index values for risk of seabird–vessel interactions by region, taxa, and season.

		Risk index							
		Summer				Fall			
Taxa	Region	Mean	Median	SD	Maximum	Mean	Median	SD	Maximum
Total seabirds	All of Alaska	13.92	11.44	10.28	70.56	13.46	12.66	8.01	69.22
	Gulf of Alaska	26.01	24.68	10.88	54.14	20.29	19.02	8.19	56.58
	Eastern Aleutians	21.38	18.66	13.07	70.56	19.71	16.15	10.26	69.22
	Northern Bering and Chukchi Seas	8.48	7.95	5.23	25.05	9.34	8.39	5.16	29.89
Auklets	All of Alaska	3.19	1.01	5.17	35.28	3.54	1.38	5.16	38.63
	Gulf of Alaska	0.88	0	1.94	9.13	1.1	0	2.62	18.65
	Eastern Aleutians	1.15	0.31	2.09	11.45	2.02	0.62	4.28	37.73
	Northern Bering and Chukchi Seas	4.72	2.39	6.31	35.28	4.61	2.08	5.95	38.63
Northern fulmars	All of Alaska	6.88	2.92	9.22	70.99	5.47	2.66	6.51	37.85
	Gulf of Alaska	9.4	8.22	9.58	30.81	6.67	6.08	6.32	23.51
	Eastern Aleutians	14.51	11.94	13.23	70.99	10.85	9.97	7.22	37.85
	Northern Bering and Chukchi Seas	2.11	1.24	2.92	24.88	1.75	0.48	3.19	25.56
Sea ducks	All of Alaska	0.76	0	5.07	91.09	1.13	0	3.91	55.21
	Gulf of Alaska	4.67	0	13.58	91.09	2.39	0	6.8	55.21
	Eastern Aleutians	0	0	0	0	0.07	0	0.39	3.33
	Northern Bering and Chukchi Seas	0.38	0	1.76	18.5	1.62	0	4.2	38.44
Shearwaters	All of Alaska	5.7	2.55	8.68	68.93	6.71	4.09	7.86	69.04
	Gulf of Alaska	5.86	1.46	8.45	38.91	7.56	3.46	8.69	31.35
	Eastern Aleutians	13.35	5.92	15.62	68.93	11.62	5.84	13.21	69.04
	Northern Bering and Chukchi Seas	3.7	2	4.65	23.95	5.4	3.88	5.34	29.87
Storm petrels	All of Alaska	3.77	0	7.34	48.35	2.33	0	5.2	47.36
	Gulf of Alaska	8.63	6.07	9.86	45.49	3.35	2.19	3.88	22.76
	Eastern Aleutians	7.59	5.8	8.42	48.35	5.08	2.65	7.24	47.36
	Northern Bering and Chukchi Seas	0.15	0	1.04	17.81	0.26	0	1.62	24.69

Better resolution of risk indices was obtained by examining total seabirds risk for the 3 focal areas. In the NGA, high risk occurred mainly in summer (Figure 4a) in PWS and near the port of Kodiak. In the EAI, high risk was identified across the north (Bering Sea) side of Unimak Pass in summer (Figure 4c) and to a lesser extent in fall (Figure 4d). In the NB‐CS, high exposure to risk occurred in summer from the Chirikov Basin through Bering Strait and into Hope Basin, and from waters off Wainwright to Point Barrow (Figure 4e). During fall, risk was similar near Bering Strait and Hope Basin but lower overall north of Icy Cape (Figure 4f).

Taxonomic groups varied in which focal regions they had highest predicted risk (Table 1). Sea ducks were present at low densities across the NB‐CS and NGA focal regions (Figure 5a,b), with moderate risk evident near Point Barrow and PWS in summer (Figure 5a) and along the coastal corridor of eastern Chukchi in fall (Figure 5b). For sea ducks, highest risk index values occurred in the NGA in summer (4.7) and fall (2.4). Auklets had the highest average risk index values in the NB‐CS region in both summer (4.7) and fall (4.6). Within this region, the highest risk for auklets was around the Bering Strait in summer and fall, with moderate risk in the northern Chukchi (Figure 5c,d). Northern fulmars had the highest risk in the EAI (14.5 in summer and 10.9 in fall), specifically near Unimak Pass both seasons (Figure 5e,f), and a broad dispersal of risk throughout the southeastern Bering Sea shelf in fall (Figure 5f). Storm petrels in fall (5.1) and shearwaters in both seasons (13.4 in summer and 11.6 in fall) also had highest risk in the EAI. Storm petrels showed high risk in the NGA in summer (8.3) (Figure 5g) and from Unimak pass to the outer Bering Sea shelf in fall (Figure 5h). Shearwaters had their highest risk along both sides of the Unimak Pass area in summer (Figure 5i) and to a lesser extent in fall (Figure 5j).

### Analyses of nighttime risk

**FIGURE 6:**
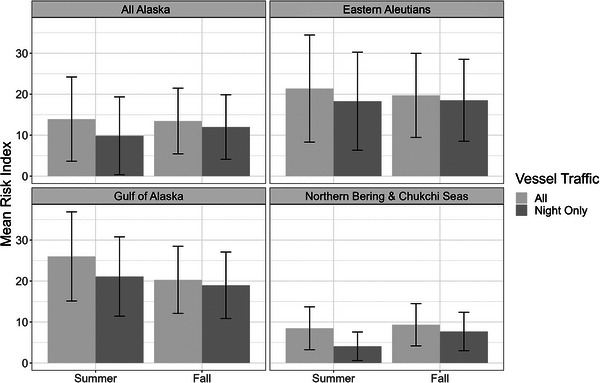
Mean index values of risk of seabird–vessel interactions for total seabirds for all vessel traffic and nighttime vessel traffic by region and season (error bars, 1 SD).

Using only data from vessels traveling at night, we found a 21% increased risk in fall compared with summer for all of Alaska. Risk from nighttime vessel traffic in the NBC‐CS focal region was especially elevated in fall, with an 88% increase over summer (Figure 6). In the EAI and NGA focal regions, risk from vessels at night was higher in summer than in fall (Figure 6). This pattern reflected the overall elevated risk in the NB‐CS region in fall compared with summer; however, the magnitude of the difference was greater for nighttime vessel traffic than for all vessel traffic combined. The higher fall risk at night also held true for auklets, sea ducks, and shearwaters at the all‐of‐Alaska scale (Appendix S5). The risk index values for storm petrels and northern fulmars were higher at night in summer than in fall, although the differences were small (Appendix S5).

## DISCUSSION

Vessel traffic poses a threat to migratory seabirds via direct encounters (e.g., collisions [Coleman et al., 2022; Merkel & Johansen, 2011]) and disturbance and displacement (Burger et al., 2019; Dehnhard et al., 2020; Dias et al., 2019; Schwemmer et al., 2011) and because it increases the potential for other threats (e.g., oil spills, chronic contamination, prey displacement, and light pollution [Humphries & Huettmann, 2014; Ryan et al., 2021]). In general, risk was greater in summer than fall for total seabirds (Table 1; Singh et al., 2015, Appendix S6). Elevated risk in summer reflects higher vessel traffic activity as well as higher average seabird density (with exception of sea ducks across all of Alaska, which exhibited higher average risk in fall) (Table 1). In addition to seasonal variations, the 3 focal regions exhibited substantial spatial differences in the distribution of risk. The EAI presented the highest average risk for total seabirds, followed by the NGA and then the NB‐CS region. In general, the distribution of risk hotspots reveals several important factors affecting the spatiotemporal distribution of seabird–vessel overlap.

First, spatial variability played a larger role than seasonal changes (at least between summer and fall) in the overall risk to seabirds at the focal region and Alaska‐wide scales. Risk was spatially clustered for all taxonomic groups, with a few areas exhibiting very high risk and many areas showing low risk. For instance, the maximum single‐cell risk index value for sea ducks in the NGA region in fall was 91.1, far higher than the cell‐level average of 4.7 (Table 1). Although we found some seasonal variability in areas of highest risk, particularly in the NGA region, there were many areas that were high risk in both summer and fall, including the Bering Strait and Unimak Pass. This consistent pattern of high‐risk areas in both seasons was due to the nonrandom distributions of both vessels and seabirds. Ships were found in the highest concentrations around ports and along established routes. When those routes intersected with established seabird colonies and the surrounding foraging grounds, there was higher exposure to risk. This finding is consistent with previous studies in the Canadian Arctic, which showed similarly consistent seasonal patterns of risk concentrated along shipping routes (Wong et al., 2018).

Both seabird and vessel distributions contributed to the spatial heterogeneity in risk. However, vessel traffic exhibited more condensed and consistent spatial patterns than did seabird distributions. For instance, areas of highest risk in PWS in the NGA region are located along established summer ferry routes (Alaska Department of Transportation & Public Facilities, 2023). In the fall, when ferry traffic decreases, the risk levels for the region decline dramatically (Figure 4a). To the west, in the EAI region, vessel traffic consists primarily of cargo and tanker vessels that operate more or less continuously along the same routes throughout the year (Sullender et al., 2021). High‐risk areas in this region, as well as in the NB‐CS region, are concentrated along vessel routes passing near seabird nesting or foraging grounds as well as locations where both vessels and seabirds funnel through narrow oceanic corridors (e.g., Unimak Pass, Bering Strait). Thus, the threat of shipping accidents—such as the 2004 grounding of the M/V *Selendang Ayu* (Kurtz, 2008)—is highest in these passes due to the proximity of land, strong ocean currents, and the high volume of oil aboard the cargo ships and tankers that frequent these routes (Sullender et al., 2021). The temporal consistency of high‐risk areas provides support for the implementation of spatial management areas.

Unlike cargo ships, tankers, and ferries, fishing vessels do not reliably follow established routes (Fletcher et al., 2020). Depending on the season, target species, and vessel type, fishing activity is more dispersed and variable than other vessel types. Although the specific routes of fishing vessels are more variable, the general areas they occupy can be fairly predictable (Taconet et al., 2019) and, in our study area, were concentrated along the continental shelf breaks in the Bering Sea and Gulf of Alaska (Figure 2). Northern fulmars and storm petrels had areas of high overlap where fishing occurs along the outer Bering Shelf. Northern fulmars have long been known to attend fishing vessels given their dietary overlap with targeted species (Wahl & Heinemann, 1979). For fulmars or other species that interact with fisheries, risk of bycatch may be higher than other vessel‐related risk types (e.g., disturbance, collision) and spatial management measures (e.g., ATBA) may not be effective measures for mitigating risk. Further, given that these seabird species and fishing vessels are often targeting the same fish species or hotspots of biological productivity, both birds and vessels are likely to follow climate change–induced shifts in biological hotspots. Therefore, although areas of elevated risk may change spatially in a changing climate, the seabird and fishing vessel overlap is likely to continue for some species.

In addition to risk along established routes and in areas of fishing activity, our results showed areas of high risk around the ports of Dutch Harbor, Kodiak, Homer, and Nome. With ports concentrating many vessels in a small amount of space, the total hours of vessel activity near port locations are disproportionately high relative to areas further offshore. Despite the high risk values we calculated near ports, the impacts of vessel traffic on seabirds in the ports themselves are qualitatively different from those of vessels at sea. Many birds avoid busy ports, although some may be disoriented by land or platform‐based bright lights at night (Gjerdrum et al., 2021). Port lights can be a problem for breeding birds that return to nearby nests at night and for fledglings disoriented during their first flights to sea (Rodríguez, Holmes, et al., 2017; Rodríguez, Moffett, et al., 2017), but this has not yet been a problem near Alaskan ports, perhaps because of remote colony locations and the long daylight hours during the main breeding season.

Beyond the spatial variability in vessel activity, we also found a high degree of variability in risk among our focal taxonomic groups, reflecting their differing life histories and feeding strategies. For example, taxa, such as shearwaters, that spend most of their time in flight likely have a lower occurrence of direct noncollision disturbance, whereas sea ducks and other species that rest or molt on the sea surface for extended periods of time may be at higher risk (Dehnhard et al., 2020). Seabird taxa, such as northern fulmars, have high dietary overlap with commercially valuable fish, which may lead to lower relative exposure to disturbance but higher rates of overlap with commercial fisheries and thus elevated risk of bycatch (Dietrich et al., 2009; Wong et al., 2014). Additionally, interindividual variability can play a role in the degree of exposure to risk, as demonstrated by the high variability in the amount of light individually tagged northern fulmars were exposed to in the Northeast Atlantic and Barents Sea (Dupuis et al., 2021).

The amount of light emitted by vessels may play an important role in the risk of detrimental seabird–vessel interactions. Light from vessels operating at night can disorient birds, causing them to fly into ships (Dick & Donaldson, 1978; Ryan et al., 2021). Brighter lights can cause more seabird disorientation and collisions (Gjerdrum et al., 2021); thus, larger vessels (e.g., LNG tankers) could pose more risk to migrating birds. We found that risk from nighttime vessel traffic was greatest in summer in the EAI and the NGA regions (Figure 6). However, in the NB‐CS region, the rapid loss of light in fall corresponded with high densities of migrating birds, increasing risk relative to summer. Seasonal sea ice cover has historically limited vessel traffic in the NB‐CS region to June through October (Frey et al., 2015; Parkinson & Cavalieri, 2008), but climate change has increased the weeks of open water (Markus et al., 2009; Stabeno & Bell, 2019). As a result, the shipping season is longer, with more traffic, particularly in the fall (Kapsar et al., 2023; Thomson et al., 2022). Thus, higher and longer vessel activity in fall coincides with migratory periods for many Subarctic‐ and Arctic‐nesting seabird species. Species like sea ducks spend more time at sea during migration and typically use over‐water routes (Day et al., 2003) and would thus be more susceptible to vessel‐based light pollution.

Sea ducks are particularly vulnerable to vessel strikes in low‐light conditions (Merkel & Johansen, 2011). Our results indicated relatively low risk of vessel–sea duck interactions, except for the Bering Strait region in fall. However, our estimate of risk for this species group was likely an underestimate for several reasons. Near‐shore seabird survey data were sparse, and very few nearshore cells had sufficient survey effort to be included in our analyses. In their analyses of sea duck collisions in Greenland, Merkel and Johansen (2011) found that 78% of collision events take place <4 km from shore. Furthermore, given that seabird surveys were exclusively conducted during daylight hours, nighttime offshore activities of sea ducks were not captured, yet radar studies at St. Lawrence Island (near Bering Strait) show that eiders tend to migrate over water and during hours of darkness (Day et al., 2003).

Across our entire study area, climate change has been driving significant shifts in the migration timing and summering areas of seabird populations (Clairbaux et al., 2019; Kuletz, Ferguson, et al., 2024; Thompson et al., 2012). As sea surface temperatures and ocean productivity increase, seabirds may begin to delay southward migration as suitable habitat stays available for longer periods of time (Patterson et al., 2021) or may travel farther northward or longitudinally to track the shifting distribution of preferred prey or open water (Kuletz, Gall, et al., 2024; Kuletz, Ferguson, et al., 2024; Lovvorn et al., 2015; Yamamoto et al., 2015). If the timing of southward migration trends later, seabirds may be exposed to more darkness and a subsequent increase in collision or disorientation caused by nighttime vessel operations, as well as risks from oil spills. However, the limited information on seabird fall migration phenology requires further research to determine whether and how changes in migration timing could alter the risk of seabird–vessel interactions.

Despite recent climate‐driven shifts in sea ice and broader ecological patterns (Danielson et al., 2020; Kapsar et al., 2023; Mueter et al., 2021), the predictability of seabird distributions and key transit routes for vessels offers opportunities to spatially and temporally target conservation measures, minimizing impacts on wildlife and marine commerce alike. Policy changes can be an effective measure for changing vessel movement patterns around sensitive areas or areas of increased risk for human safety (Huntington et al., 2019; Smith, 2007; Sullender et al., 2021). Recommended routing measures and areas to be avoided (ATBAs) were implemented around the Aleutian Islands in 2016 and, more recently, in the Bering Strait in 2019 (IMO, 2014, 2017). The Aleutian Islands ATBAs have been highly effective, with upward of 88% of voyages in compliance with the routes (Sullender et al., 2021). Additionally, requirements for certain light types (e.g., red lights) could minimize light pollution effects during nighttime travel (Syposz et al., 2021), and, in the fishing industry, bycatch mitigation strategies, such as bird scaring lines, help minimize interactions between birds attracted to fishing vessel activities (Tamini et al., 2023). (For an example, see recommendations for best practices for collision mitigation at https://www.fws.gov/service/technical‐assistance‐prevent‐bird‐vessel‐strike‐alaska‐marine‐environment.) Although fishing and research vessels may be obligated to follow best operating practices in sensitive environments and respond to US *Endangered Species Act* section 7(a)(2) consultations to avoid negative seabird–vessel interactions, other types of activity (e.g., tourism and shipping) are not so obligated.

Although we have provided an important baseline for evaluating seabird–vessel overlap and thus risk of interactions and contamination, there is a need to better understand the impacts of this overlap, including population‐level effects. There is a lack of published data on the rates of seabird–vessel interactions, and what data exist are primarily limited to small samples (e.g., Merkel & Johansen, 2011) and anecdotal reports (e.g., Dick & Donaldson, 1978). However, reports of seabird–vessel interactions describe incidents involving anywhere from several to several thousand birds colliding with individual vessels and frequently emphasize the poor weather and/or low‐light conditions (Black, 2005; Dick & Donaldson, 1978; Merkel & Johansen, 2011; Montevecchi, 2005). The risk of vessel accidents, often resulting in oil spills, varies with ship type, and studies suggest accidents increase with vessel speed and age, and under darkness, poor weather conditions, or the presence of ice (Bye & Aalberg, 2018; Rezaee et al., 2016). Given the increased potential for seabird–vessel overlap in the fall (a period with rapidly decreasing light and increased storm activities) in the northern portion of the study area, there is a need to better understand the rates of seabird–vessel conflicts in order to estimate population‐level consequences. Likely of greater consequence is the risk of chronic pollution (Wiese & Robertson, 2004) and catastrophic contamination from vessel activity or accidents. Catastrophic seabird mortality was recorded during the *Exxon Valdez* oil spill in Alaska (Piatt et al., 1990) and is a conservation concern worldwide (Helm et al., 2015).

Our quantification of risk was confined to the boundaries of the study area itself and to distribution of seabird surveys in it. For instance, upper Cook Inlet did not have sufficient seabird survey effort to be included in our analysis, yet is home to the Port of Anchorage, one of the busiest ports in Alaska (McDowell Group, 2020). In addition to regular tanker visits and the corresponding risk of catastrophically high‐volume oil spills, vessel traffic in Cook Inlet is dominated by tugboats and fishing vessels targeting salmon, with relatively low risk to birdlife (Fletcher et al., 2021). Russian waters were also almost entirely excluded from our analyses due to a paucity of seabird surveys, yet the Chukotka Peninsula hosts important concentrations of several sea duck species (Smith et al., 2017). Russian vessel traffic in fall has increased in the Gulf of Anadyr and northeastern Chukotka Peninsula in recent years (Kapsar et al., 2023), which may present emerging hotspots of seabird–vessel risk.

The Bering Strait corridor is used by Russia, the United States, and other countries, and economic and political incentives for increased use of Arctic passages require new management decisions that incorporate wildlife habitat uses (Brigham, 2011; Lovvorn, et al. 2009). Nearshore areas immediately surrounding seabird breeding colonies may be particularly sensitive to vessel impacts but were not well represented in the seabird database. Although most surveys could not include waters adjacent to seabird colonies, the at‐sea data clearly showed colony‐location effects, with halos of higher seabird densities within a couple hundred kilometers of large colony sites. Thus, the influence of colony location was indirectly incorporated into the overlap analyses. There may be regions of high risk not identified by this analysis but that are nonetheless important to consider when designing and implementing management approaches. Future research approaches that allow for transboundary and nearshore mapping of seabird distributions (e.g., telemetry) could be especially useful techniques to help fill knowledge gaps.

Although we included several taxa of conservation concern in our analyses, the potential impacts of interactions with vessel activities on a given seabird species is highly dependent upon the population size. For taxa with large populations, such as northern fulmars, even relatively severe impacts from vessel traffic (e.g., bird storms, oil spills) may not have substantial population‐level impacts. However, for rare species (e.g., Kittlitz's murrelet [*Brachyramphus brevirostris*]) or species restricted to specific areas (e.g., whiskered auklets [*Aethia pygmaea]*), the same level of overlap with vessel activity, and associated risks, could have detrimental population‐level effects. The actual quantification of impacts to seabirds from vessel activity depends on many factors beyond the scope of this analysis and outstanding primary data needs. In addition to variability in life history and feeding strategy mentioned previously, the frequency and severity of different impacts could also depend on other factors, such as weather patterns (e.g., for artificial lights) or ship safety standards and ship characteristics (e.g., for risk of oil spill). Although risk values with respect to population impacts are taxa and threat specific, our results provide a baseline for locations and seasons where these risks can be anticipated. In the case of wildlife managers, who frequently must make holistic decisions based on multiple overlapping threats, broadscale analyses such as ours help prioritize resources in areas of highest conservation need.

## Supporting information



Additional supporting information may be found in the online version of the article at the publisher's website.
