# Supplementary material for: A multiscale seasonal examination of the risk of harm to seabirds from vessels based on co‐occurrence in Alaskan waters
Source: Conserv Biol. 2025 Aug 20;40(1):e70115. doi: 10.1111/cobi.70115 (PMC12856794; doi:10.1111/cobi.70115)
Supplement: Supplementary file 1 — Additional supporting information may be found in the online version of the article at the publisher's website. [file COBI-40-e70115-s001.docx]

**Supporting Information**

Additional supporting information may be found in the online version of the article at the publisher’s website.

Appendix S1. List of species in each taxonomic group.


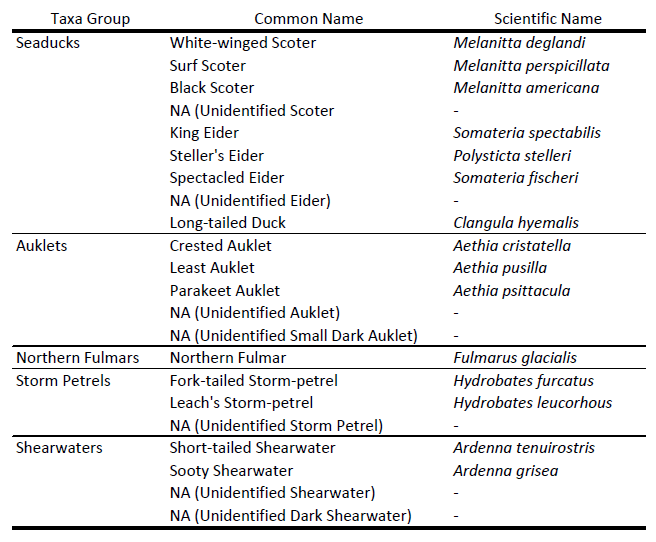


Appendix S2. List of species in each taxonomica group.

| **Taxa** | **Type of interaction** | **Reference** |
| --- | --- | --- |
| Seaducks | Vessel disturbance to molting eiders | Dehnhard et al. 2020 |
|  | Vessel disturbance (flushing) for 4 sea duck species | Schwemmer et al. 2011 |
|  | Light attraction caused bird strikes (eiders) | Merkel & Johansen 2011 |
|  | Offshore platform light attraction causes bird strikes, alters behavior | Ronconi et al. 2015 (review) |
|  | Attraction to fishing vessels | Wahl & Heinemann 1979 |
|  | Vessel strike mortality | NMFS 2020 |
|  | Bycatch in gillnet fisheries | Dietrich et al. 2025 (Alaska); Žydelis et al. 2013 (review) |
|  | Oil spill mortality | Piatt et al. 1990 |
| Auklets | Light attraction caused bird strikes (crested auklets) | Dick & Donaldson 1978 |
|  | Bycatch in gillnet fisheries | Dietrich et al. 2025 (Alaska); Žydelis et al. 2013 (review) |
|  | Oil spill mortality | Piatt et al. 1990 |
| N. Fulmars | Light attracts to fishing vessels; alters behavior | Dupuis et al. 2021 |
|  | Light attraction caused bird strikes (coastal, offshore platforms, vessels) | Gjerdrum et al. 2021 |
|  | Offshore platform light attraction causes bird strikes, alters behavior | Ronconi et al. 2015 (review) |
|  | Attraction to fishing vessels | Wahl & Heinemann 1979 |
|  | Bycatch in Alaska longline fisheries | Dietrich et al. 2009 |
|  | Bycatch in gillnet fisheries | Žydelis et al. 201 (review) |
|  | Oil spill mortality | Piatt et al. 1990 |
| Storm petrels | Light causes disorientation | Montevecchi 2005 (review) |
|  | Light attraction caused bird strikes | Montevecchi 2005 (review); Coleman et al. 2022; Ryan et al. 2021 |
|  | Light attraction caused bird strikes (coastal, offshore platforms, vessels) | Gjerdrum et al. 2021 |
|  | Offshore platform light attraction causes bird strikes, alters behavior | Ronconi et al. 2015 (review) |
|  | light-induced mortality on land | Rodríguez et al. 2017a |
|  | Attraction to fishing vessels | Wahl & Heinemann 1979 |
|  | Bycatch in gillnet fisheries | Žydelis et al. 2013 (review) |
| Shearwaters | Light causes disorientation | Montevecchi 2005 (review) |
|  | Light attraction caused bird strikes (coastal, offshore platforms, vessels) | Gjerdrum et al. 2021 |
|  | Offshore platform light attraction causes bird strikes, alters behavior | Ronconi et al. 2015 (review) |
|  | Light-induced mortality on land | Rodríguez et al. 2017a |
|  | Attraction to fishing vessels | Wahl & Heinemann 1979 |
|  | Bycatch in Alaska longline fisheries | Dietrich et al. 2009 |
|  | Bycatch in gillnet fisheries | Dietrich et al. 2025 (Alaska); Žydelis et al. 2013 (review) |
|  | Oil spill mortality | Piatt et al. 1990 |

Appendix S3. Summary of vessel traffic intensity and seabird survey effort by region.


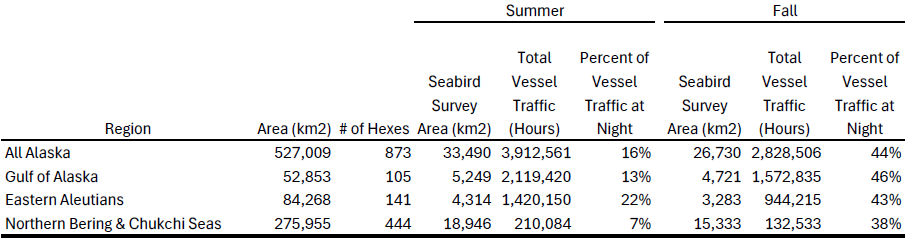


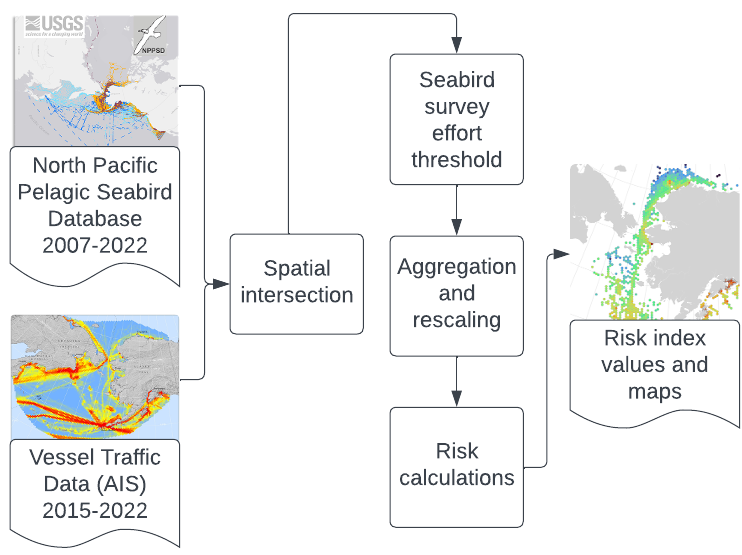


Appendix S4. Workflow diagram illustrating steps in the analysis of seabird-vessel traffic interaction risk.


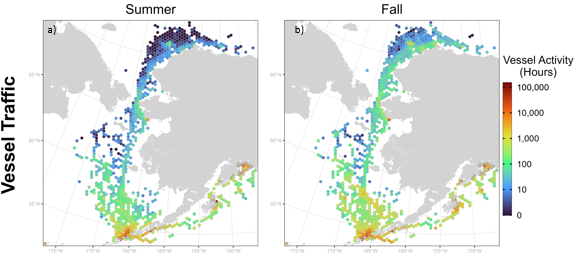


Appendix S5. Map of Alaska-wide nighttime vessel traffic in (a) summer and (b) fall.


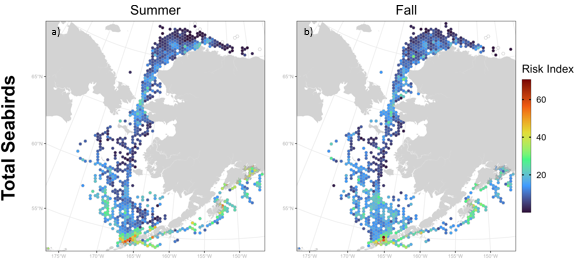


Appendix S6. Seasonal Risk Index maps of seabird-vessel interactions for all seabird species.


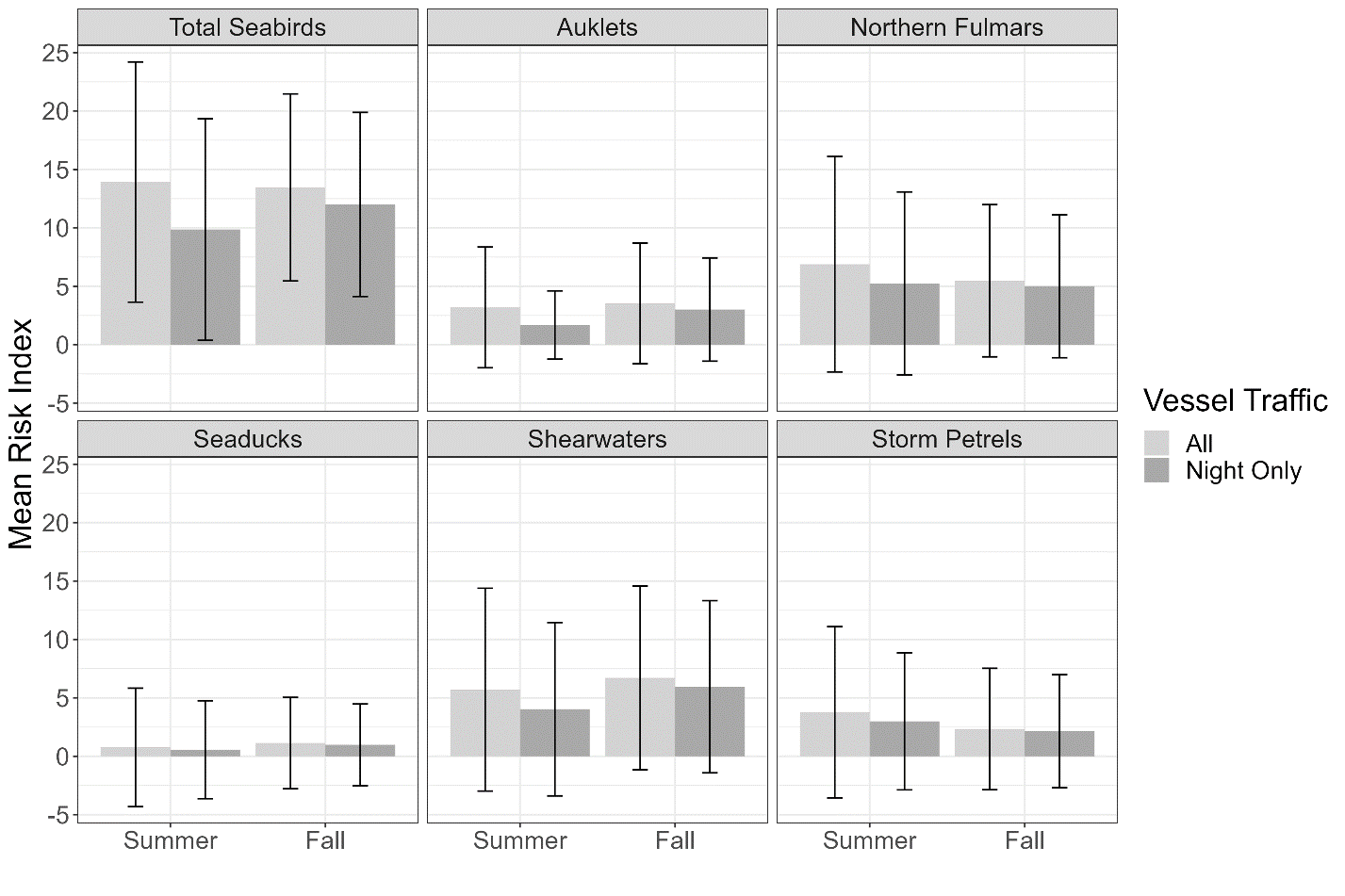


Appendix S7. Mean risk index value for seabird-vessel interactions in the study region. Light gray bars indicate all vessel traffic was included in the analysis while dark gray bars indicate only nighttime vessel traffic was analyzed. Error bars depict +/- 1 SD.
